# Possible Mechanism of Sucrose and Trehalose-6-Phosphate in Regulating the Secondary Flower on the Strong Upright Spring Shoots of Blueberry Planted in Greenhouse

**DOI:** 10.3390/plants13172350

**Published:** 2024-08-23

**Authors:** Hui-Ling Wu, Sui-Lin Zhang, Xin Feng, Ya-Qian Zhang, Bing-Jie Zhou, Man Cao, Ya-Ping Wang, Bao-Shi Guo, Zhi-Xia Hou

**Affiliations:** State Key Laboratory of Efficient Production of Forest Resources, Key Laboratory for Silviculture and Conservation of Ministry of Education, Blueberry Research & Development Center, Beijing Forestry University, Beijing 100083, China; wuhl1998@163.com (H.-L.W.); lg20170404@bjfu.edu.cn (S.-L.Z.); feng0504xin@163.com (X.F.); zyqaixuexi@163.com (Y.-Q.Z.); zhoubj0705@163.com (B.-J.Z.); caoman2016@163.com (M.C.); wypinging@163.com (Y.-P.W.); guobaoshi@sdu.edu.cn (B.-S.G.)

**Keywords:** *Vaccinium corymbosum* L., flower bud differentiation, spring shoots, sucrose, trehalose-6-phosphate, *VcTPS*

## Abstract

Secondary flowering is the phenomenon in which a tree blooms twice or more times a year. Along with the development of blueberry (*Vaccinium corymbosum* L.) fruits in spring, a large number of secondary flowers on the strong upright spring shoots were noticed in blueberries planted in the greenhouse. To reveal the cause and possible regulatory mechanism of the phenomenon, we clarified the phenological characteristics of flower bud differentiation and development on the spring shoots by combining phenological phenotype with anatomical observation. Furthermore, the changes in carbohydrates, trehalose-6-phosphate (Tre6P), and the relationship among the key enzyme regulatory genes for Tre6P metabolism and the key regulatory genes for flower formation during the differentiation process of apical buds and axillary buds were investigated. The results showed that the process of flower bud differentiation and flowering of apical and axillary buds was consistent, accompanied by a large amount of carbohydrate consumption. This process was positively correlated with the expression trends of *VcTPS1/2*, *VcSnRK1*, *VcFT*, *VcLFY2*, *VcSPL43*, *VcAP1*, and *VcDAM* in general, and negatively correlated with that of *VcTPP*. In addition, there is a certain difference in the differentiation progress of flower buds between the apical and axillary buds. Compared with axillary buds, apical buds had higher contents of sucrose, fructose, glucose, Tre6P, and higher expression levels of *VcTPS2*, *VcFT*, *VcSPL43*, and *VcAP1*. Moreover, *VcTPS1* and *VcTPS2* were more closely related to the physiological substances (sucrose and Tre6P) in axillary bud and apical bud differentiation, respectively. It was suggested that sucrose and trehalose-6-phosphate play a crucial role in promoting flower bud differentiation in strong upright spring shoots, and *VcTPS1* and *VcTPS2* might play a central role in these activities. Our study provided substantial sight for further study on the mechanism of multiple flowering of blueberries and laid a foundation for the regulation and utilization of the phenomenon of multiple flowering in a growing season of perennial woody plants.

## 1. Introduction

Flower bud differentiation is crucial for reproduction and economic fruit harvest, especially for perennial woody fruiting tree species. Naturally, most subtropical and temperate deciduous perennial woody plants (apples, peaches, pears, etc.) need two consecutive growing seasons to finish the course from flower bud differentiation to flowering [1,2]. Flower buds usually need a certain chilling requirement to flower after bud differentiation is complete [3]. In recent years, the phenomenon attracted increasing attention that the second or even multiple bud differentiation and flowering in addition to the first flower set in some woody perennial trees with relatively stable seasonal flowering, such as chestnuts [4], blueberries [5], pears [6], *Magnolia × soulangeana* [7,8], and grapes [9]. These flower bud differentiation and flowering other than the first bloom in the same growing season can be called secondary flowers [4]. These flower buds can flower immediately after their differentiation is complete, without any chilling requirements. Secondary flowering has both advantages and disadvantages in fruit production, which may improve economic benefits or affect the normal growth of trees. Therefore, it is very important to understand the regulation mechanism of secondary flowers in order to use them scientifically.

Carbohydrates are important for flower bud differentiation and flowering [10,11], which can serve both as energy and signal transmitters [11], and sucrose is the main sugar signal in plants. The metabolic balance between starch and sucrose is also crucial for flower induction in plants [12]. Importantly, sugar-mediated flower differentiation involves the signal metabolite trehalose-6-phosphate (Tre6P) [13,14]. Tre6P levels are closely related to sucrose levels in plant vegetative tissues and stem apical meristems, which can be seen as a representative of sucrose status in plants [15]. In *Arabidopsis*, Tre6P mainly induces flowering by regulating the expression of flowering integration genes *FLOWERING LOCUS T* (*FT*) and *TWIN SISTER OF FT* (*TSF*) in leaves and apical meristems, while also influencing the expression of the flowering pattern gene *SQUAMOSA PROMOTER BINDING PROTEIN-LIKE* (*SPL*), which affects the age pathway to induce flowering [16]. In apples, exogenous sucrose significantly increases the expression of *MdTPS1/2/4/10/11* and *MdFT1* in flower buds, thereby promoting flowering [17]. SNF1-related kinase 1 (SnRK1) is an evolutionarily conserved energy-sensing protein kinase. It can regulate plant stomatal development and enhance plant environmental adaptability by sensing changes in carbohydrate metabolism [18]. In *Arabidopsis*, Tre6Ps regulate the development of *tps1* mutants by inhibiting the expression of *SnRK1* [19]. Meanwhile, the Tre6P level in plants is also regulated by TREHALOSE-6-PHOSPHASE SYNTHESIS (TPS) and TREHALOSE-6-PHOSPHASE PHOSPHASE (TPP) [20]. In *Magnolia liliiflora* Desr. “Hongyuanbao”, the expression levels of *MlTPS1/5/6/7/9* are significantly higher in the secondary flower bud differentiation than that of the primary flower. In addition, exogenous sucrose shortens the time to complete flower bud differentiation by activating *MlTPS1/5* [10]. In *Prunus sibirica*, the expression level of *PsTPPF* in flower buds of late-flowering apricots is much higher than that of early-flowering apricots, indicating that *PsTPPF* inhibits their flowering process.

*FT*, *LEAFY* (*LFY*)*,* and *APETALA1* (*AP1*) are considered key genes in flower regulation [21]. The transition of flowering period (including dormancy) is controlled by flowering genes *FT* and *TFL*, *miR156/SPL* and *miR172/AP2* modules, as well as *DORMANCY-ASSOCIATED MADS BOX* genes (*DAM*) in response to external environmental conditions [21]. *FT*s promote the flowering process of blueberries by activating the expression of the downstream flowering genes *LFY*, *SOC1*, and *AP1* [22]. Overexpressing *VpSBP11* from grape could significantly upregulate *LFY* expression, leading to an early flowering phenotype in *Arabidopsis* [23]. *AP1*s are key genes that control calyx and carpel formation and petal development [21]. The expression of *MawuAP1* in *Magnolia wufengensis* could not restore the sepal and petal formation in *Arabidopsis ap1* mutants [24]. In peaches, the expression of *PbDAM5/6* is upregulated during floral differentiation and downregulated during floral organ enlargement [25].

**Figure 1:**
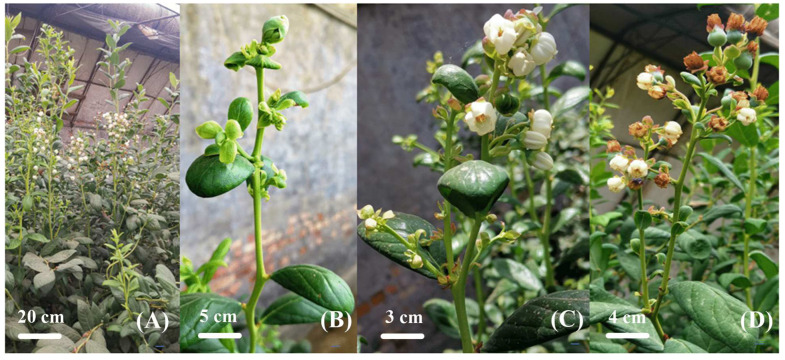
Blueberry plants secondary flower directly after the differentiation of flower buds in strong upright spring shoots. (**A**) The growth and flowering of strong upright spring shoots on blueberry plants. (**B**) The flower buds have already sprouted on the strong upright spring shoots. (**C**) Flowers bloom on the upper part of strong upright spring shoots. (**D**) Flowering and setting of blueberries on the strong upright spring shoots.

Normally, blueberries are clear seasonal flowering perennial woody deciduous fruit trees. However, flower bud initiation in the southern highbush blueberry cv. O’Neal occurred twice during the growing season without chill hours was reported [26]. Meanwhile, in our research, lots of flower buds formed and directly blossomed on the spring shoots of blueberries cultivated in greenhouses in north China in spring, as soon as their differentiation was completed in late spring (April) (Figure 1). In our study, along with the primary fruit from green to maturity on the greenhouse-cultivated blueberries in April and May, a large number of secondary flowers appeared on the strong upright spring shoots. Different from previous reports, this kind of blueberry secondary flower shows great advantage in the number of flower buds and concentration of the formation periods, which can form good-quality secondary fruit. It has the potential to create a huge opportunity for greenhouse blueberry spring production of secondary fruit to supply the market. However, there is also the risk that the yield and quality of the primary blueberry fruit in spring might be affected due to poor management coordination. How to make good use of this phenomenon is a fascinating and challenging study. Therefore, it is crucial to clarify the differentiation reasons and regulatory mechanisms of such flower buds.

Considering the importance of carbohydrate metabolism for flower bud differentiation and bloom, and based on the phenological characteristics of flower bud differentiation and the development of apical buds and axillary buds on the strong upright shoots, the changes in carbohydrates and trehalose-6-phosphate (Tre6P) were studied, respectively. The relationship between the above changes and the key enzyme regulatory genes for Tre6P metabolism (*VcTPS*s, *VcTPP*, and *VcSnRK1*) and the key regulatory genes for flower formation (*VcFT*, *VcLFY2*, *VcSPL43*, *VcAP1*, and *VcDAM*) during the differentiation process of apical and axillary buds was investigated. Fortunately, this study makes full use of the time difference in the flower bud differentiation process between apical and axillary buds to analyze the secondary flowering mechanism separately, further ensuring the reliability of the development period and process. This not only lays a favorable foundation for the involvement of sugar signaling in regulating the molecular mechanisms of flowering in woody plants but also provides a reference for management measures in production.

## 2. Results

### 2.1. Flower Buds Differentiation Characteristics on Strong Upright Spring Shoots of Blueberry

**Figure 2:**
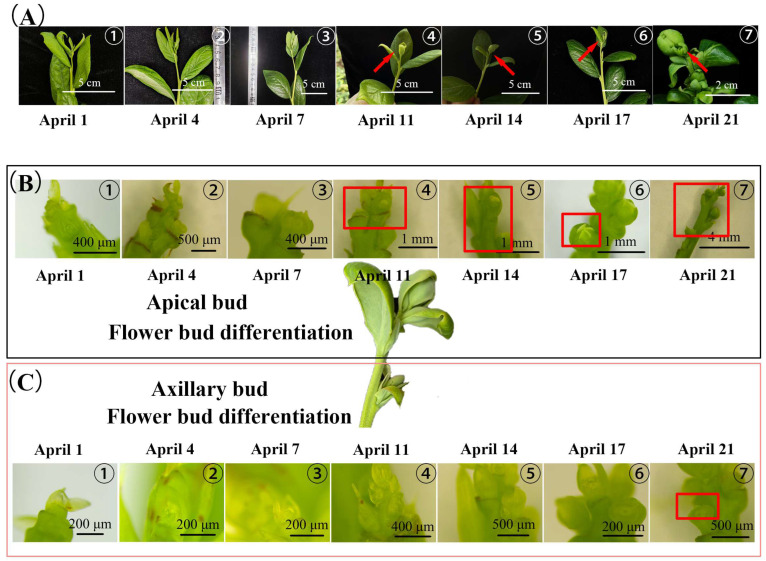
The external morphological characteristics and internal meristem changes during the differentiation process of strong upright spring shoots apical and axillary buds of blueberries (1–21 April 2022). (**A**) The external morphological characteristics of strong upright spring shoots. ①~⑦ represent April 1st, 4th, 7th, 11th, 14th, 17th, and 21st, respectively. The red arrows on ④~⑦ emphasize the process by which the outer bracts of the apical bud gradually open (similar to flower bud germination). (**B**) Microscopic photos of apical meristem tissues during each sampling. (**C**) Microscopic photos of axillary bud meristem tissues during each sampling. (**B**,**C**) indicate that the differentiation process of apical buds was similar to that of axillary buds, but the terminal bud differentiation process was significantly earlier than that of axillary buds by about 10 days. The red rectangle indicates where white petal tissue can be seen in the meristem tissue.

**Figure 3:**
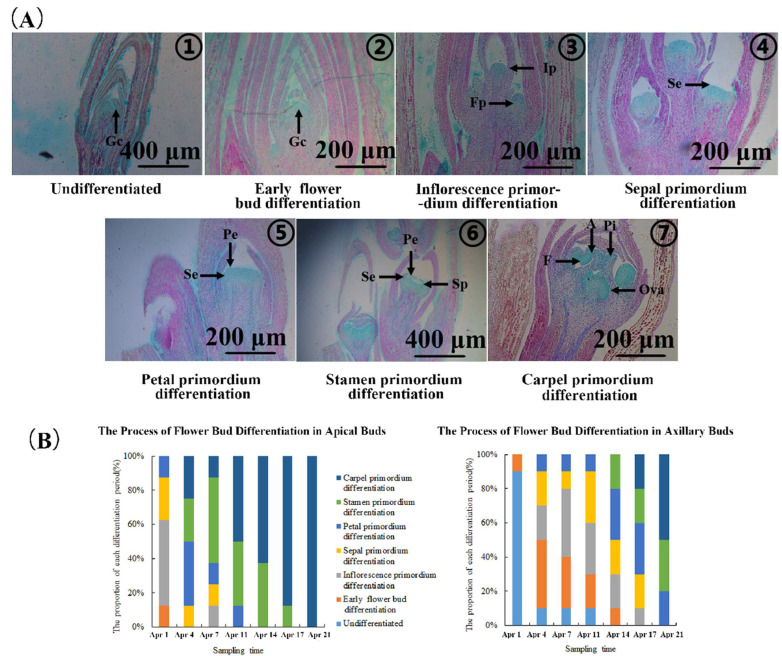
The process of flower bud differentiation in the apical and axillary buds of strong upright spring shoots of blueberries. (**A**) Anatomical structural characteristics of blueberry strong upright spring shoot flower bud differentiation at different stages. ① Undifferentiated flower buds; ② Early flower bud differentiation stage; ③ Inflorescence primordium differentiation stage; ④ Sepal primordium differentiation stage; ⑤ Petal primordium differentiation stage; ⑥ Stamen primordium differentiation stage; ⑦ Carpel primordium differentiation stage. Gc (growth cone); Ip (inflorescence primordium); Fp (flower primordium); Se (sepal primordium); Pe (petal primordium); Sp (stamen primordium); A (anthers); F (filament); Pi (pistil); Ova (ovary). (**B**) The proportion of samples taken at each developmental stage is based on the differentiation period of apical and axillary buds in (**A**).

In late April to May 2022, lots of flowers and secondary fruits could be seen on the strong upright spring shoots of blueberries planted in the greenhouse, while the primary spring fruits were ripening (Figure 1). They developed rapidly and stood upright, and almost no obvious flower bud stage could be noticed, which was markedly different from that of the primary flower buds, which remained on the branch for a long time before blossoming (Figure 1 and Figure 2A–C). We also observed the same phenomenon in 2021 (Table S3). In 2021, 47.95% of strong upright spring shoots finally bloomed (Table S3-1). In addition, the fruiting branches of strong upright spring shoots accounted for 51.81% of the total number of branches (Table S3-2). The number of fruits of strong upright spring shoots accounted for 76.98% of the total. The fruit weight of strong upright spring shoots accounted for 77.95% of the total. Therefore, in 2022, we further observed and statistically analyzed the flower formation process of apical and axillary flower buds of strong upright spring shoots in blueberries (Figure 2 and Figure 3). There are also very few strong upright spring shoots without flower buds; about 13.33% of strong upright shoots will continue to elongate their apical buds, and their axillary buds will tiller (Figure 1A and Table S3-1).

In about 20 days, the first 5 or so nodes of the strong upright spring shoots transitioned from vegetative growth to a flowering state. The differentiation process of apical buds was similar to that of axillary buds, but the terminal bud differentiation process was significantly earlier than that of axillary buds by about 10 days (Figure 2B,C and Figure 3B). For apical buds, on April 1st, 50% of buds were in the inflorescence primordium differentiation stage. On April 4th, approximately 37.5% of buds developed to the petal primordium differentiation stage. On April 7th, 50% of buds were in the stamen primordium differentiation stage. On April 11th, 50% of buds began to differentiate into carpel primordium. Starting from April 11th, the proportion of carpel primordium differentiation in apical buds gradually increased. By April 21st, almost 100% of buds had completed carpel primordium differentiation. The appearance showed that the bracts of apical buds wrapped around the middle meristem, forming elliptical apical buds on April 11th (Figure 2B). Subsequently, the bracts of apical buds would slowly open as apical buds developed, rather than turning dark brown over time like the bracts formed in autumn [27]. According to Spiers’s description [28] of blueberry flower buds differentiation and flowering process, apical buds on April 11th were in state 1 (Figure 2A(④): no signs of buds enlargement, bracts surrounding inflorescence), apical buds on April 14th and April 17th were in state 2 (Figure 2A(⑤,⑥): visible bud enlargement, bracts separated, but inflorescence not visible), and apical buds on April 21st were in state 3 (Figure 2A(⑦): bracts separated, top of flowers visible). It was believed that after April 11th, apical buds began to germinate. Following, the large-scale flowering and fruiting will be seen in succession, as shown in Figure 1C,D.

At the same time, the process of axillary bud differentiation, which was generally consistent with that of apical buds, was ongoing (Figure 2A). On April 1st, approximately 90% of axillary buds were in the undifferentiated stage (Figure 3B). On April 4th, 40% of axillary buds proceeded to the flower initiation stage. On April 7th, 40% of axillary buds entered the inflorescence primordial differentiation stage, and 30% of axillary buds were still in the flower initiation stage. On April 11th, the numbers of axillary buds in sepal and inflorescence primordia were both about 30%. On April 14th, about 30% of axillary buds were in the petal primordium differentiation stage and 20% of axillary buds were in the stamen primordium differentiation stage. On April 17th, about 30% of axillary buds were in the petal primordium differentiation stage, and about 20% of axillary buds had already undergone the carpel primordium differentiation stage. As of April 21st, more than 50% of axillary buds had finished carpel primordium differentiation.

### 2.2. The Spatiotemporal Changes of Carbohydrates during Flower Bud Differentiation

**Figure 4:**
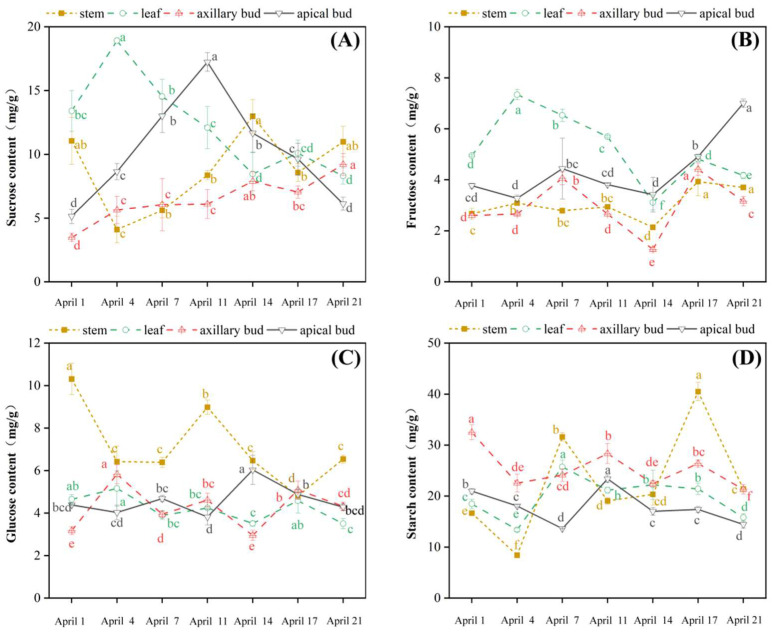
Carbohydrate content in different parts of blueberry spring shoots (apical buds, axillary buds, leaves, and stems) during flower bud differentiation. (**A**–**D**) represent the contents of sucrose, fructose, glucose, and starch in blueberries, respectively. The significant differences in carbohydrate content between different stages of the same tissue were represented by lowercase letters (values = means ± SE, Duncan’s test, *n* = 3).

The sucrose content of apical buds showed an overall trend of first increasing and then decreasing (Figure 4A). In the flower organ primordium differentiation stage (April 1st–11th), sucrose content in apical buds significantly increased and peaked in the carpel primordium differentiation stage (April 11th), where it was 2.4 times higher than that of the inflorescence primordium differentiation stage (April 1st). When apical buds were in the germination stage (April 11th–21st), their sucrose content significantly decreased. At the same time, sucrose content in axillary buds showed an overall dynamic increase, reaching its highest level in the carpel primordium differentiation stage (April 21st), but significantly lower than that of the sucrose level in apical buds in the carpel primordium differentiation stage (April 11th). During the differentiation process, sucrose content in apical buds was generally higher than that in axillary buds, except for April 21st. The change in sucrose content in leaves significantly increased in the early stage of axillary bud flowering differentiation (April 4th) and then decreased until April 14th, followed by increasing on April 17th, then decreasing to the lower level on the carpel primordium differentiation stage (April 21st). However, the trend of sucrose content in stems was opposite to that in leaves. The inflection point appeared consistently, indicating that there might be some transport relationship between sucrose in leaves and stems.

Fructose content in apical buds was relatively stable in the flower organ differentiation stage (April 1st–11th) and significantly increased in the germination stage (April 14th–21st) (Figure 4B). The changing trend of fructose content in axillary buds was almost synchronized with that in apical buds, and it was significantly lower than that in apical buds in all the differentiation stages. There were significant increases in the axillary bud flower initiation stage (April 7th) and carpel primordium differentiation stage, but significant decreases in the early flower organ formation stage (April 14th) and carpel primordium differentiation stage (April 21st), respectively. The changing trend of fructose contents in leaves was synchronized with that of sucrose content. Fructose content in stems was relatively stable in the early stage of axillary bud undifferentiation, reached its lowest point in the flower organ petal and stamen primordium differentiation stage (April 14th), then significantly increased in the carpel primordium differentiation stage (April 14th–21st).

Glucose content in apical buds was relatively stable in the flower bud differentiation stage (April 1st–21th) (Figure 4C), but increased significantly when flower buds sprouted (April 14th). The changes in glucose content both in axillary buds and leaves were consistent. Their contents increased in the flower initiation stage of axillary buds (April 4th), then decreased in the early stage of flower organ primordium differentiation (April 4th–14th), and increased in the late stage of flower organ primordium differentiation (April 17th–21st). The glucose level in stems was significantly higher than that in other organs, showing a fluctuating downward trend overall. In the early stage of axillary bud flower initiation (April 1st–4th), the glucose level in stems sharply decreased and then significantly increased on April 11th. In the axillary flower organ primordium differentiation stage (April 11th–21st), glucose content increased on April 11th and significantly decreased.

In apical buds, starch content decreased in the early stage of flower organ primordium differentiation (April 1st–7th) (Figure 4D). While in the carpel primordium differentiation stage (April 11th), it significantly increased to the peak (April 11th) and gradually decreased with the germination of apical buds (April 11th–21st). The starch contents of axillary buds were significantly higher than those of apical buds, and their overall change trend showed a fluctuating downward trend that was synchronized with that of apical buds. The changing trend of starch content in leaves and stems was consistent, with a large fluctuation in the whole stage of flowering differentiation. In the early stage of axillary flower differentiation (April 4th), starch content in axillary buds and leaves sharply decreased to the lowest value, then significantly increased on April 7th, and then showed a significant downward trend on April 11th. Moreover, starch content peaked on April 17th.

### 2.3. Tre6P Content and the Related Genes’ Expression during Flower Bud Differentiation

**Figure 5:**
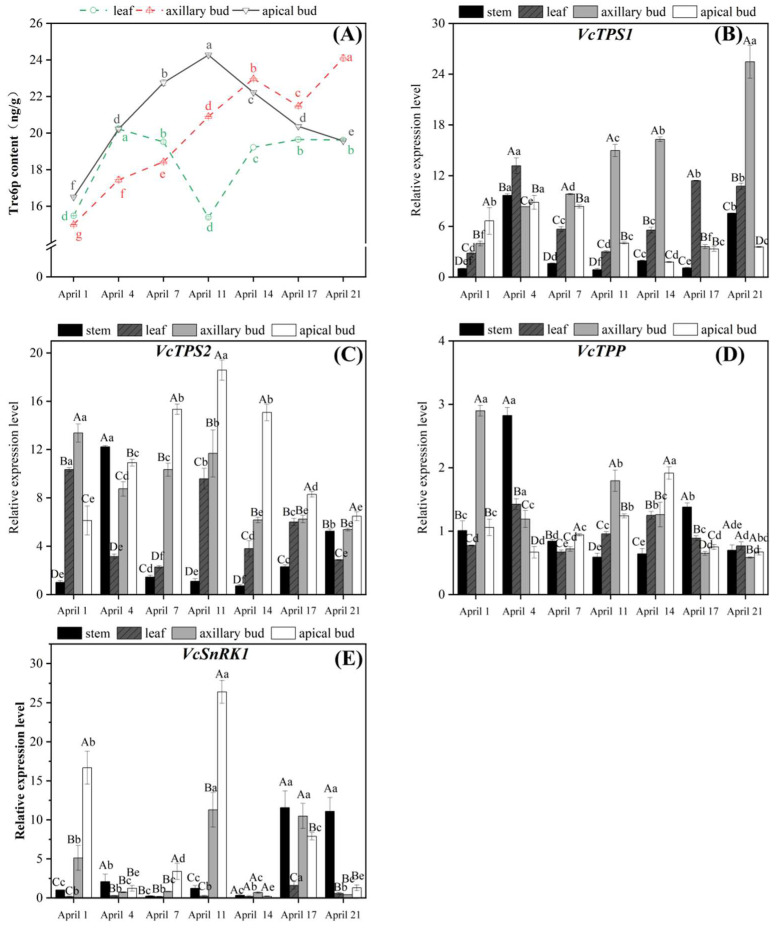
Tre6P content and the expression patterns of related genes in strong upright spring shoots of blueberries. (**A**) Tre6P content in strong upright spring shoots of blueberries. (**B**–**E**) Changes in expression levels of *VcTPS1*, *VcTPS2*, *VcTPP*, and *VcSnRK1* in strong upright spring shoots of blueberries. The significance of gene expression differences between different tissues during the same period was represented by uppercase letters, while the significance of gene expression differences between different periods of the same organization was represented by lowercase letters (values = means ± SE, Duncan’s test, *n* = 3).

Trehalose-6-phosphate (Tre6P) content in apical buds significantly increased in the flower organ primordium differentiation stage (April 1st–11th), which increased by approximately 50% in the carpel primordium differentiation stage (April 11th). Subsequently, its content significantly decreased, dropping by about 20% by April 21st (Figure 5A). In the flower bud differentiation stage (April 1st–21st), the Tre6P content of axillary buds generally showed an upward trend. On April 21st, the Tre6P content in axillary buds increased by approximately 55% compared to its flower induction stage (April 1st). The change trends of Tre6P and sucrose content in apical and axillary buds were synchronous (Figure 3A and Figure 4A). In the flower organ primordium differentiation stage (April 1st–11th in apical), the Tre6P content of apical buds was higher than that of axillary buds. Tre6P content in leaves significantly increased and peaked in the flower initiation stage of axillary buds (April 1st–4th), then decreased and reached a valley on April 11th. Afterward, it significantly increased in the axillary flower organ primordium differentiation stage (April 11th–21st).

The *VcTPS1* expression in apical buds showed a roughly increasing and then decreasing trend (Figure 5B). In the early stage of flower organ differentiation (April 1st–7th), its expression significantly increased and peaked. During the process of apical bud germination (April 11th–21st), *VcTPS1* expression was significantly downregulated and maintained at a relatively low level. Overall, the *VcTPS1* expression in axillary buds was significantly higher than in other parts and increased throughout the process except for the pistil primordium differentiation stage (April 17th), where its expression was significantly downregulated. The changes in *VcTPS1* were consistent with those of the Tre6P content from April 1st to 17th (Figure 4A,B). The overall change trend of *VcTPS1* expression in leaves was also consistent with that of Tre6P content. However, *VcTPS1* expression in stems was only significantly upregulated on April 4th and April 21st, and relatively lower at other stages.

The *VcTPS2* expression in apical buds was significantly higher than in other parts (excluding April 1st), which trend was highly consistent with that of Tre6P and sucrose contents (Figure 4A and Figure 5A,C). *VcTPS2* expression peaked in apical buds when apical buds were in the carpal primordium differentiation stage (April 11th), then significantly downregulated. The overall expressions of *VcTPS2* in axillary buds were stepwise downward, with significantly upregulation in the early stage of flower organ primordium differentiation (April 7th–11th). *VcTPS2* expression in leaves was high in the axillary flower induction stage (April 1st), then significantly downregulated in the axillary flower initiation stage (April 4th–7th), followed by significant upregulation on April 11th. After a sharp downregulation on April 14th, its expression stabilized at a relatively low level. The *VcTPS2* expression in stems was significantly upregulated on April 4th and April 21st, while it was relatively low in other stages.

The *VcTPP* expression in apical buds was maintained relatively low in the flower organ primordium differentiation stage (April 1st–11th) and significantly upregulated in the germination stage (April 11th–14th) (Figure 5D). Then it was significantly downregulated and maintained at a lower level as the germination of apical buds. *VcTPP* expressed relatively high in axillary buds in the flower initiation stage (April 1st). *VcTPP* expression in axillary buds was significantly downregulated during its flower bud initiation stage (April 4th–7th) and significantly upregulated in the early stage of the flower organ primordium differentiation stage (April 11th), followed by a continuous decline. Compared with other organs, *VcTPP* expression in leaves was relatively low and remained relatively stable. On April 4th and April 14th, its expression was significantly upregulated. The *VcTPP* expression in stems was significantly upregulated only on April 4th and was lower in other stages.

It could be seen that *VcSnRK1* was mainly expressed in apical buds, axillary buds, and stems (Figure 5E). In apical buds, *VcSnRK1* expression was high in the early stage of flower organ primordium differentiation (April 1st), then downregulated on April 4th and April 7th, and peaked in the carpel primordium differentiation stage (April 11th), then significantly downregulated. The *VcSnRK1* expression in axillary buds showed a similar trend and an upregulated point, respectively, in the early stages of flower organ primordium differentiation (April 11th) and carpel primordium differentiation (April 17th). From April 11th to 14th, its expression was significantly downregulated. The *VcSnRK1* expression in leaves was relatively low, showing an overall trend of first increasing and then decreasing. On April 17th, *VcSnRK1* expression was highest in leaves but much lower than in other parts. The *VcSnRK1* expression in stems was low in the early stages of flower differentiation, then significantly upregulated, and peaked on April 17th and 21st.

### 2.4. Expression of Flower Related Genes during Flower Bud Differentiation

**Figure 6:**
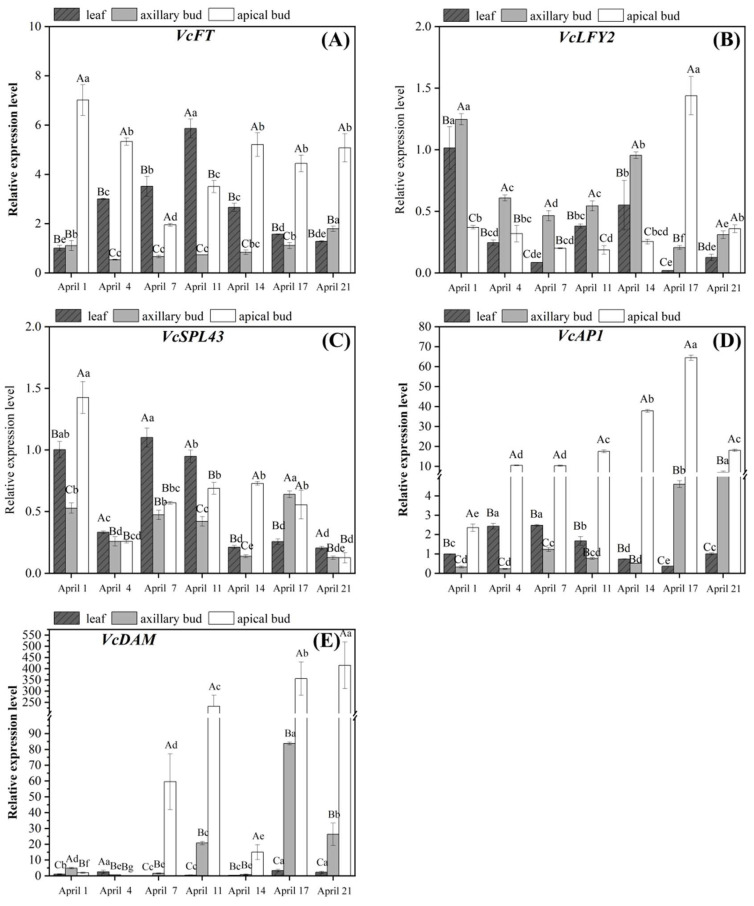
Expression patterns of flowering-related genes in strong upright spring shoots of blueberries. (**A**–**E**) Changes in expression levels of *VcFT*, *VcLFY2*, *VcSPL43*, *VcAP1*, and *VcDAM* in strong upright spring shoots of blueberries. The significance of gene expression differences between different tissues during the same period was represented by uppercase letters, while the significance of gene expression differences between different periods of the same organization was represented by lowercase letters (values = means ± SE, Duncan’s test, *n* = 3).

*VcFT* was mainly expressed in apical buds and leaves (Figure 6A). *VcFT* expression in apical buds was highest in the inflorescence primordium differentiation stage (April 1st) and significantly downregulated in the early stage of flower organ primordium differentiation (April 4th–7th). Subsequently, *VcFT* expression in apical buds was significantly upregulated as apical buds turned to germinate (April 14th) and stabilized at relatively high levels in the flower bud germination stage (April 14th–21st). The *VcFT* expression in axillary buds was significantly lower than that in apical buds. It was high on April 1st (flower induction stage), then significantly downregulated in the flower initiation stage (April 4th), and then significantly upregulated from the flower organ primordium differentiation stage to the carpel primordium differentiation stage (April 11th–21st). The *VcFT* expression in leaves significantly increased from April 1st to the highest level on April 11th, followed by a significant decrease.

The *VcLFY2* expression in apical buds was low from April 1st to 14th but significantly upregulated on April 17th (Figure 6B). However, at this time, the *VcLFY2* expression in axillary buds and leaves decreased to the lowest level. On April 21st, its expression was significantly downregulated. The *VcLFY2* expression in axillary buds and leaves was significantly downregulated in the flower initiation stage (April 1st–7th) and significantly upregulated in the early stage of the flower organ primordium differentiation stage (April 11th–14th). Subsequently, its expression in axillary buds and leaves was significantly downregulated on April 17th and then upregulated again in the carpel primordium differentiation stage (April 21st).

The *VcSPL43* expression in apical buds was relatively high on April 1st and then significantly downregulated (Figure 6C). In the apical flower organ primordium differentiation stage (April 4th–11th), its expression gradually increased and remained at a high level in the apical bud germination stage (April 11th–17th), before gradually decreasing to the lowest level. The *VcSPL43* expression in axillary buds and leaves was similar from April 1st to 14th. Its expression was significantly downregulated and then upregulated in the flower initiation stage (April 1st–7th) and significantly downregulated in the early stage of flower organ differentiation (April 11th–14th). Its expression in axillary buds was significantly upregulated and reached its peak on April 17th before significantly decreasing. However, its expression in leaves had no significant changes from April 14th to 21st.

The *VcAP1* expression was significantly higher in apical buds than in other parts, followed by axillary buds (Figure 6D). The *VcAP1* expression in apical buds was significantly upregulated from April 1st to April 17th. On April 21st, its expression was significantly downregulated. *VcAP1* expression in axillary buds was relatively low in the flower induction stage and the early stage of the flower organ differentiation stage (April 1st–14th). It was significantly upregulated in the late stage of the flower organ differentiation stage (April 17th–21st) and upregulated to the highest level in the carpel primordium differentiation stage (April 21st). Although its expression was still lower than that in apical buds at this stage, it was significantly upregulated by about 22 times from April 1st to 21st. When axillary buds were in the flower induction stage (April 1st–7th), *VcAP1* expression in leaves was higher than that in the flower organ differentiation stage (April 11th–21st), but its expression was lower than that in apical and axillary buds.

The *VcDAM* expression in apical buds showed an upward trend and was significantly upregulated in the flower organ primordium differentiation stage (April 1st–11th) (Figure 6E). When apical buds turned to germinate (April 14th), *VcDAM* expression sharply downregulated to approximately 6% of the expression on April 11th. In the apical bud germination stage (April 17th–21st), *VcDAM* expression significantly increased and reached its highest value on April 21st. *VcDAM* expression in axillary buds also showed an upward trend, but its expression was significantly lower than that in apical buds. In the flower induction stage and the early stage of flower organ differentiation (April 1st–11th), its expression was significantly upregulated, followed by a sharp downregulation. In the late stage of flower organ primordium differentiation (April 17th–21st), its expression was significantly upregulated and reached its peak on April 17th. The *VcDAM* expression in leaves was significantly upregulated on April 4th, then significantly downregulated and maintained at a lower level. In the late stage of flower organ primordium differentiation (April 17th–21st), its expression was significantly upregulated, consistent with the changing trend of *VcDAM* expression in axillary buds.

### 2.5. Correlation Analysis among Carbohydrate and Tre6P Related Genes and Flowering Genes

**Figure 7:**
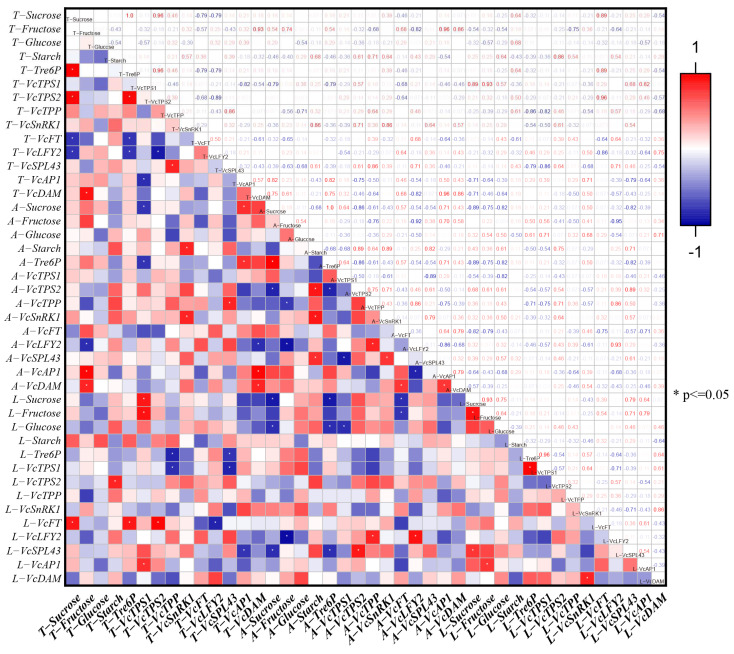
Correlation analysis of carbohydrate content and related gene expression levels. This is a correlation analysis of carbohydrate content and related gene expression levels between the apical buds, axillary buds, and adjacent leaves during the differentiation process of strong upright spring shoots of blueberries. T, A, and L represent apical buds, axillary buds, and adjacent leaves, respectively. The correlation analysis and significance test were conducted using the Spearman method, and * represents the significance level *p* ≤ 0.05. The red blocks and numbers represented the positive correlation, while the blue blocks and numbers represented the negative correlation.

In order to further reveal the possible mechanism by which carbohydrates (sucrose and Tre6P) regulate the secondary flowering of the strong upright spring shoots in blueberries, the correlation among carbohydrate content and gene expression in apical buds, axillary buds, and leaves was analyzed (Figure 7). In apical buds, sucrose contents, Tre6P contents, and *VcTPS2* expression were significantly positively correlated with each other. However, Tre6P content was negatively correlated with *VcFT* expression, but the correlation was relatively lower. Moreover, *VcTPS1* expression was positively correlated with *VcAP1* expression. In axillary buds, Tre6P content and sucrose content were significantly positively correlated. Tre6P content and *VcTPS1* expression were positively correlated, while Tre6P content and sucrose content were positively correlated with *VcTPS2* expression. Starch content, *VcSnRK1* expression, *VcTPS1,* and *VcSPL43* expression were negatively correlated. In addition, *VcTPP* and *VcLFY2* expression were positively correlated. In leaves, Tre6P content was positively correlated with *VcTPS1* expression, but the correlation was relatively lower. In addition, *VcSnRK1* expression was significantly positively correlated with *VcDAM* expression.

At the same time, there was a certain correlation between carbohydrate contents and the related gene expression in apical buds, axillary buds, and leaves. Starch content in apical buds was significantly positively correlated with *VcTPS2* expression in leaves. Tre6P content and *VcTPS2* expression in apical buds were significantly positively correlated with *VcFT* expression in leaves. *VcTPS1* expression in apical buds was positively correlated with sucrose content and *VcAP1* expression in leaves but negatively correlated with Tre6P content in axillary buds. The *VcTPP* expression in apical buds was negatively correlated with Tre6P content and *VcTPS1* expression in leaves. *VcSnRK1* expression in apical buds was positively correlated with starch content, *VcSnRK1* expression in axillary buds, and *VcTPS2* expression in leaves. *VcSPL43* expression in apical buds was positively correlated with *VcTPP* expression in axillary buds. However, it was negatively correlated with *VcTPS1* expression and Tre6P content in leaves. *VcAP1* expression in apical buds was positively correlated with Tre6P content in axillary buds in leaves. *VcDAM* expression in apical buds was positively correlated with *VcAP1* and *VcDAM* expression in axillary buds, but it was negatively correlated with *VcLFY2* expression in axillary buds. Starch content in axillary buds was positively correlated with *VcTPS2* expression and *VcSnRK1* expression in apical buds. *VcTPS2* expression in axillary buds was significantly positively correlated with *VcSPL43* expression in leaves. *VcLFY2* expression in axillary buds and leaves was positively correlated.

## 3. Discussion

Most of the seasonal flowering perennial woody deciduous plants need two consecutive growth seasons for their flower bud differentiation to blossom. Fruit trees in the Rosaceae family (apples and pears) form flower buds in autumn and winter and enter a dormant state before sprouting and flowering in the following spring [2,4]. The phenomenon in seasonal flowering trees that appeared as soon as flower bud differentiation finished has gradually attracted attention, such as chestnuts and grapes [9]. They all have secondary flower bud differentiation and flowering besides the primary flowering during the same growing season. In this study, the blueberry variety “M7” planted in a greenhouse experienced two large numbers of concentrated flower bud differentiation, respectively, in spring and autumn within one growing season. Flower buds formed on strong upright spring shoots in spring exhibited characteristics of flowering as soon as differentiation finished without undergoing a natural dormancy stage (Figure 1).

Both flower bud differentiation and germination require energy consumption, accompanied by the decomposition of a large amount of carbohydrates [29]. Sucrose, Tre6P, and *TPS* have important promoting effects on the flower formation, floral organ development, and flowering of plants [10,13,30]. Sucrose and Tre6P contents, as well as *VcTPS2* expression in apical buds and *VcTPS1* expression in axillary buds, all reached their peak in their carpel primordium differentiation stage on strong upright spring shoots of blueberries. During this process, the *VcTPS2* expression in apical buds and the *VcTPS1* expression in axillary buds were synchronized with the changes in sucrose and Tre6P contents (Figure 4A and Figure 5A–C). And sucrose, Tre6P contents, and *VcTPS2* expression in apical buds were significantly positively correlated with each other, while sucrose, Tre6P contents, and *VcTPS1* expression in axillary buds were significantly positively correlated with each other (Figure 7). This indicated a close relationship among sucrose, Tre6P contents, and *VcTPS1/2* expression, and they could promote direct flowering on strong upright spring shoots after flower bud differentiation. In *Magnolia grandiflora*, after exogenous 60 mM Tre6P, *MlTPS1/5* expression is significantly upregulated in the mid-stage (25 days) and late-stage (40 days) of flower bud differentiation. *MlTPS1* expression and *MlTPS5* expression are, respectively, higher during the secondary and primary flower bud differentiation processes [10]. During the daytime, Tre6P in the leaves of the source organ regulates sucrose levels by affecting sucrose synthesis. At night, Tre6P in the leaves regulates the transient breakdown of starch, which is used to supply the demand for sucrose in flower bud differentiation and flowering [31]. In *Arabidopsis*, even with high Tre6P content, the lack of sucrose signal makes it unable to flower normally [32]. This further demonstrates the importance of sucrose and Tre6P in the flower bud differentiation and flowering process. The *VcSnRK1* expression in apical buds and axillary buds was significantly positively correlated with starch content in axillary buds (Figure 7). In transgenic sweet potatoes, the overexpression of *IbSnRK1* upregulates the expression of genes related to starch biosynthesis pathways, enhancing the activity of key enzymes involved in starch synthesis and thereby increasing starch content [33]. In *Arabidopsis*, Tre6P has been identified as a conformational inhibitor of *SnRK1* [34]. Sucrose is converted into Tre6P under the action of *TPS1*, thereby regulating the flowering of *Arabidopsis* plants [13]. The *VcTPP* expression in apical buds was significantly negatively correlated with Tre6P content and *VcTPS1* expression in leaves (Figure 7). This further indicated that there was an extremely complex regulatory relationship among starch, sucrose, Tre6P, *VcTPS1/2*, *VcTPP*, and *VcSnRK1* in the different parts during the flower bud differentiation and flowering process in strong upright spring shoots of blueberries.

*VcFT*, *VcLFY2*, *VcSPL43*, *VcAP1*, and *VcDAM* also played important roles in the flower bud differentiation and flowering process of strong upright spring shoots of blueberries (Figure 6A–E). The *VcFT* expression was relatively high in the germination stage of apical buds (April 14th–21st). The *VcLFY2* expression peaked in the apical bud bract separation stage (April 17th). The expression level of *VcSPL43* showed a rough upward trend both in the differentiation process of apical and axillary buds (Figure 6C). In *Arabidopsis*, *FT* is expressed in the companion cells of leaves, and then the FT protein is transported from leaves to SAM through sieve tubes, promoting its flowering [35]. In figs (*Ficus carica)* , the *FcLFY* expression is higher in apical buds, and the vegetative growth time of *Arabidopsis* plants overexpressing *FcLFY* is significantly shortened, and their flowering time is advanced [36]. The *VcAP1* expression continued to increase in the apical and axillary bud differentiation stage (April 1st–17th) and peaked before germination (Figure 6D). *AP1* is significantly upregulated with the development of the rose floral organ differentiation phase [37]. The *VcDAM* expression remained relatively high in the flower organ primordium differentiation and germination stages, both in apical and axillary buds (Figure 5E). However, in Japanese apricots, the *PmDAM1-6* expression is relatively high during the flower bud dormancy and significantly downregulated when the dormancy is released [38]. This indicated that *VcDAM* might play different roles, which depend on the species and stages of flower bud development. The *VcFT* expression in leaves was significantly positively correlated with sucrose content, Tre6P content, and *VcTPS2* expression in apical buds. The *VcSPL43* expression in leaves was significantly positively correlated with *VcTPS2* expression in axillary buds, and *VcAP1* expression in apical buds, sucrose content, and Tre6P content in axillary buds were significantly positively correlated. The *VcDAM* expression in leaves was significantly positively correlated with *VcSnRK1* expression (Figure 7). In *Arabidopsis*, *AtTPS1* can regulate the transmission of sucrose signals to plant meristems and activate the expression of *AtFT*. In *Magnolia* treated with 60 mM Tre6P, *MlFT* and *MlLFY* expression are higher in the late stage of flower bud differentiation (35 days), and the *MlAP1* expression is higher in the mid-stage of flower bud differentiation (30 days) [10]. This indicated that *VcFT*, *VcLFY2*, and *VcAP1* might mainly play a promoting role in the germination stage of strong upright spring shoots of blueberries and were closely related to Tre6P content and *VcTPS2* and *VcTPP* expression. The *VcLFY2* expression was significantly upregulated in the early stage of flower organ differentiation (April 14th) (Figure 6A,B). *VcSPL43*, *VcAP1*, and *VcDAM* expression was significantly upregulated in the late stage of flower organ differentiation (April 17th) (Figure 6C–E). This indicated that *VcLFY2* probably played the promoting role in the flower induction stage and the early stage of flower organ primordium differentiation in axillary buds, while *VcSPL43*, *VcAP1*, and *VcDAM* probably played the promoting role in the late stage of flower organ primordium differentiation. Therefore, it was speculated that *VcSnRK1* might positively regulate starch content, and *VcTPS1/2* might promote the expression of *VcFT*, *VcLFY2*, *VcSPL43*, *VcAP1*, and *VcDAM* by regulating the conversion of sucrose to Tre6P, enabling the strong upright spring shoots of blueberries to blossom directly without endo-dormancy after differentiation. Meanwhile, *VcTPS1/2* and *VcTPP* jointly regulated Tre6P content, which might also cause starch to decompose into sucrose, thereby increasing sucrose levels in flower buds.

There was a certain difference in the differentiation process between apical buds and axillary buds. This difference may be due to differences in carbohydrate content and related gene expression levels. Sucrose, Tre6P contents, and *VcTPS2/1* expression peaked on April 11th in apical buds, while on April 21st in axillary buds, which were their carpel primordium differentiation stage, respectively (Figure 4 and Figure 5). That is to say, sucrose and Tre6P might create synergies in flower bud differentiation. This phenomenon conforms to the characteristics of apical dominance [39,40,41]. In addition, differentiation process regulation was more closely related to *VcTPS2* in the apical buds and was more closely related to *VcTPS1* in the axillary buds (Figure 7). In rice, *OsNAC23* increases Tre6P content by inhibiting the expression of *OsTPP1*, thereby promoting the distribution of carbohydrates from source organs to sink organs to maintain carbohydrate balance [42]. The sucrose content in leaves was significantly negatively correlated with sucrose contents and Tre6P content in axillary buds (Figure 7). It was suggested that sucrose transport might exist among the leaves and buds, and the decomposition of sucrose in leaves might be conducive to its transport to the buds, thereby promoting the differentiation of flower buds in the blueberry.

In addition, *VcFT*, *VcAP1*, and *VcDAM* expression in apical buds were significantly higher than those in axillary buds, and sucrose and glucose contents in stems were significantly higher than those in axillary buds (Figure 4 and Figure 5). In the early stage of flower bud differentiation (April 1st–14th), the *VcLFY2* expression in axillary buds was significantly higher than that in apical buds (in flower organ primordium differentiation) (Figure 6B). At the same time, sucrose, fructose, and Tre6P contents in leaves continued to decrease during flower bud differentiation (Figure 4A,B and Figure 5A). Carbohydrates stored in plants can be transported from leaves to flower buds or flowers [43]. In walnuts, the growth of flower buds depends on the long-distance transportation of carbohydrates, which is achieved through the interaction between the xylem and phloem [44]. In addition, the starch content of apical buds was significantly lower than that of axillary buds (Figure 4D), possibly due to the rapid development of apical buds leading to the rapid decomposition of starch. When tomato plants face a short-term environment of insufficient light, starch in flower organs begins to decompose, exerting a self-protection mechanism and providing energy for the growth and development of the flower organs, thereby alleviating the impact of insufficient light on flower development [14]. Therefore, it was speculated that blueberry leaves served as a powerful “reservoir” for carbohydrate metabolism, and the presence of apical dominance led to the preferential transport of nutrients from leaves to apical buds through stems, resulting in the inhibition of sucrose, fructose, and Tre6P content, as well as the expression levels of *VcFT*, *VcAP1*, and *VcDAM* in axillary buds, thereby promoting preferential rapid differentiation and flowering of apical buds.

**Figure 8:**
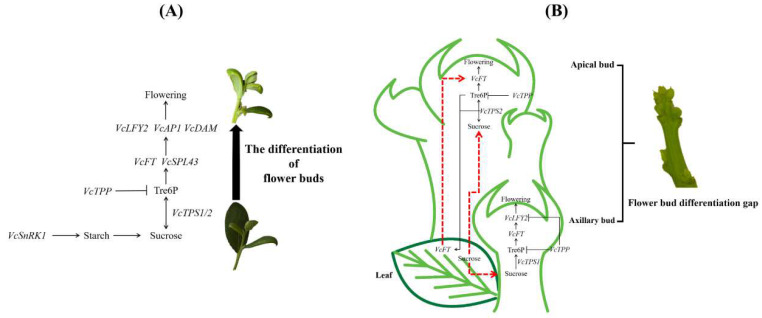
(**A**) A regulatory model for the seasonal germination of blueberry strong upright spring shoot flower buds. (**B**) Differences in the regulation of flower bud differentiation between blueberry strong upright spring shoot apical and axillary buds. The red dotted arrows represent unknown transportation relationships, the black arrows represent positive regulatory relationships, and black T-bar represents negative regulatory relationship.

In summary, this study proposed a possible regulatory pathway for carbohydrates to regulate flower bud differentiation and flowering on strong upright spring shoots of blueberries in the greenhouse (Figure 8). Carbohydrate contents (sucrose, fructose, glucose, starch, and Tre6P) in apical and axillary buds on strong upright spring shoots of blueberries decreased from flower bud differentiation to flowering, providing energy for their differentiation and flowering process. *VcSnRK1* positively regulated the synthesis of starch, which was further decomposed into sucrose. Sucrose was converted to Tre6P under the action of *VcTPS1/2*, which promoted the *VcFT* expression in leaves. The *VcFT* protein was transported to apical buds through sieve tubes of stems. *VcFT* and *VcSPL43* activated the expression of downstream flower-related genes *VcLFY2*, *VcAP1*, and *VcDAM*, promoting direct flowering as soon as the flower bud differentiated. Tre6P levels were also inhibited by *VcTPP*. In addition, *VcTPS1* mainly played a role in axillary buds, while *VcTPS2* mainly played a role in apical buds. There was a significant positive correlation between *VcTPS2* in apical buds and *VcFT* in leaves and between sucrose, Tre6P, and *VcTPS2* in apical buds. There was a positive correlation between sucrose, Tre6P, and *VcTPS1* in axillary buds. Further exploration and validation are needed to investigate the interactions among sucrose, Tre6P, and key genes involved in the metabolism pathways of Tre6P (*VcTPS*s, *VcTPP*, and *VcSnRK1*), as well as key genes involved in flower regulation (*VcFT*, *VcLFY2*, *VcSPL43*, *VcAP1*, and *VcDAM*), and the direct flowering process on the blueberry strong upright spring shoots flower bud differentiation in blueberries.

## 4. Materials and Methods

### 4.1. Plant Materials

Highbush blueberries (*Vaccinium corymbosum* “M7”, 7 years old) cultivated in the greenhouse in the blueberry plantations in the eastern suburbs of Beijing. There are 38 rows of blueberry plants in the experimental greenhouse. In 2021, we investigated the strong upright spring shoots of all blueberry plants in the greenhouse and counted the number of strong upright spring shoots that finally bloomed and the number of fruiting branches. In April 2022, we selected 21 rows in the middle of the experimental shed, with every seven rows being one experimental area, for a total of three experimental areas. To avoid interference from other factors, 200 strong upright spring shoots with consistent growth conditions are randomly selected as experimental materials in each experimental area. Thirty typical strong upright spring shoots in the experimental area are marked as observation samples for statistical analysis of strong upright spring shoots that finally bloomed. The flowering rate of strong upright spring shoots in 2021 is the proportion of strong upright spring shoots that have bloomed in the entire experimental greenhouse. The flowering rate of strong upright spring shoots in 2022 is the proportion of strong upright spring shoots that have bloomed in 30 markedly strong upright spring shoots. The labeled apical buds (the apex node of new shoots), axillary buds (the second, third, and fourth nodes from the apex of new shoots), leaves, and stems on nodes with axillary buds were collected every 3–4 days. Twenty apical buds and twenty axillary buds were randomly selected, observed under the stereomicroscope, and photos taken, then fixed with FAA for microscopic observation. All experimental materials were collected at 9:00~11:00 in the morning. The remaining materials were frozen in liquid nitrogen and stored at −80 °C for future analysis. Then the frozen blueberry samples were used for further analysis, including measuring sucrose, fructose, glucose, starch, Tre6P content, and RNA extraction.

### 4.2. Microscopic Observation of Flower Buds Differentiation Process

Referring to the method of Marasek Ciolakowska [45], the fixed samples were subjected to gradient alcohol dehydration, waxing, embedding, trimming, sectioning, sectioning, and staining. Photos were taken and observed under a biological microscope, and the number of flower buds in the differentiation process of all apical and axillary buds on each date was counted.

### 4.3. Soluble Sugars, Starch, and Tre6P Content Analyses

#### 4.3.1. Sucrose, Fructose, and Glucose Content

According to the method of Ito [46], 0.1 g of frozen blueberry sample was taken for soluble sugar extraction. Four milliliters of an 80% (*v*/*v*) alcohol solution was added, followed by a 40 min water bath at 80 °C. The supernatant was collected by centrifugation at 5000× *g* for 10 min at room temperature. Then, 2 mL of an 80% alcohol solution was added to the precipitate, and the above steps were repeated for extraction twice. After merging the supernatant, it was decolorized with activated carbon, filtered, and precipitated. Finally, 80% alcohol was added to make up to 10 mL. The determination of sucrose and fructose content in blueberry tissue refers to the method [47], using the hydrochloric acid resorcinol method. The glucose content determination method follows the steps of the glucose (GLU) assay kit, and the OD value of the sample is measured at a wavelength of 505 nm.

#### 4.3.2. Starch Content

Starch extraction follows the method of Zhang [48], with slight modifications. After extracting soluble sugars, 2 mL of distilled water was added to the precipitate. After a 15 min water bath at 80 °C, 2 mL of a 9.2 mol/L perchloric acid solution was added after the liquid was cooled to room temperature. After shaking and extracting for 15 min, 5000× *g* was centrifuged at room temperature for 15 min. The supernatant was taken, and 2 mL of a 4.6 mol/L perchloric acid solution was added to the precipitate. After shaking and extracting for 15 min, the supernatant was collected again by centrifugation. Finally, the precipitate was washed with 2 mL of distilled water, and all the supernatants were combined to 10 mL. The starch content was determined using the anthrone sulfuric acid method, and the OD value of the sample was measured at a wavelength of 534 nm.

#### 4.3.3. Tre6P Content

The method for extracting Tre6P content refers to Xu [49], with slight modifications. The mass fraction of the mixed solution of blueberry tissue powder (about 0.1 g) and PBS solution was 10%. The mixed solution was vortexed at room temperature for 15 s and extracted for 30 min at 4 °C. The prepared homogenate was centrifuged at 3000× *g* for 15 min at 4 °C, and the supernatant was taken. The determination method followed the steps of the plant trehalose-6-phosphate ELISA kit (Yan Qi Biological Technology, Shanghai, China). After adding the termination solution, the OD value of the sample was measured at a wavelength of 450 nm within 15 min.

### 4.4. RNA Extraction and RT-qPCR

RT-qPCR was used to detect the expression levels of fucose metabolism and flowering related genes during blueberry flower bud differentiation. RNA samples were isolated from blueberry stems, leaves, apical buds, and axillary buds using the method of Feng [27]. The HIScript lll All-in-One RT SuperMix Perfect for qPCR Kit (Vazyme, Beijing, China) was used to reverse-transcribe total RNA into cDNA. The qPCR reaction was performed using the Taq Pro Universal SYBR qPCR Master Mix Kit. The premix was prepared according to the reagent kit method, and the RT instrument program method was set to the SYRB method. The data analysis adopted the 2^−ΔΔCt^ method to calculate relative gene expression levels, with *VcUBC28* as the internal reference gene to standardize the data. The primers and gene IDs for all genes are shown in Table S1. The CDS sequences of all genes are sourced from the GDV database (https://www.vaccinium.org accessed on 20 August 2022). The relative expression levels of all genes are shown in Table S2.

### 4.5. Data Statistical Analysis

Three biological replicates were set up for each experimental group. IBM SPSS Statistics 26 was used to calculate the mean and standard deviation of sample content and expression level, and a one-way ANOVA was used for the same group of data. Duncan’s test indicates significant differences at *p* < 0.05. The correlation analysis was conducted using the Spearman method. The data for the correlation analysis were based on the average of the above measurement indicators. Pictures were drawn using Origin 2023 software.

## 5. Conclusions

The flower bud differentiation on strong upright spring shoots of blueberries planted in the greenhouse showed rapid differentiation and flowering directly as soon as the differentiation finished. The apical bud flower differentiation process was the same as the axillary buds, while the proceeding was about 10 days earlier. In apical buds, axillary buds, leaves, and stems, sucrose, fructose, glucose, starch, and trehalose-6-phosphate (Tre6P) contents, especially sucrose and Tre6P contents, changed synchronously with the flower development process of apical and axillary buds. The change in carbohydrate content during flower development was significantly correlated with the high expression of *VcTPS1/2*, *VcSnRK1*, *VcFT*, *VcLFY2*, *VcSPL43*, *VcAP1*, *VcDAM*, and the low expression of *VcTPP*, respectively. Moreover, sucrose and Tre6P contents and the expression levels of *VcTPS2*, *VcSnRK1*, *VcFT*, *VcAP1,* and *VcDAM* in apical buds were higher than those in axillary buds, which might be the reasons for the faster flower bud differentiation of apical buds. The flower bud differentiation processes of apical and axillary buds were more closely related to *VcTPS2* and *VcTPS1*, respectively. These results lay a theoretical foundation for revealing the molecular regulatory mechanism of direct flowering without dormancy after the differentiation of strong upright spring shoots of blueberries.

## Data Availability

Data are contained in the article.

## Supplementary Materials

The following supporting information can be downloaded at: https://www.mdpi.com/article/10.3390/plants13172350/s1, Table S1: Primer sequences for 9 genes in this study; Table S2: The average value and standard error of the relative expression levels of all genes. Table S3-1: The proportion of strong upright spring shoots that finally bloomed in 2021 and 2022. Table S3-2: Production and Characteristics of Blueberry Secondary Fruits in Sunlight Greenhouses in 2021.
